# Methotrexate oral or subcutaneous for rheumatoid arthritis (MOOSE): study protocol for a multi-centre randomised trial

**DOI:** 10.1186/s13063-025-09020-4

**Published:** 2025-11-12

**Authors:** Sarah McClure, Rebecca Haydock, Yuanfei Su, Caroline Rick, Steven Blackburn, Michael Brooks, Christopher J. Edwards, Christian Mallen, Karim Raza, Clare Jinks, Matt Stevenson, Alan Montgomery, Trish Hepburn, Abhishek Abhishek

**Affiliations:** 1 Nottingham Clinical Trials Unit, The University of Nottingham, Nottingham, UK; 2 University of Birmingham, Birmingham, UK; 3 PPI Co-applicant, The University of Nottingham, Nottingham, UK; 4 Midlands Partnership University NHS Foundation Trust, Stafford, UK; 5 NIHR Southampton Clinical Research Facility, University Hospital of Southampton Clinical Research Facility, Southampton, UK; 6 Keele University, Keele, UK; 7 Department of Rheumatology, Bronglais Hospital, Hywel Dda University Health Board, Carmarthen, Wales UK; 8 Sheffield Centre of Health and Related Research, The University of Sheffield, Sheffield, UK; 9 The University of Nottingham, Nottingham, UK

**Keywords:** Randomised controlled trial, Methotrexate, Oral, Subcutaneous, Rheumatoid arthritis

## Abstract

**Background:**

Rheumatoid arthritis is the commonest chronic inflammatory arthritis. Oral methotrexate is recommended as the first-line disease modifying drug for its management, and subcutaneous injections are typically prescribed if there is gastrointestinal intolerance or suboptimal efficacy. It is not known whether subcutaneous methotrexate is more effective and cost-effective compared to oral methotrexate when used as first-line treatment in people diagnosed with rheumatoid arthritis. The Methotrexate Oral Or SubcutanEous (MOOSE) trial aims to compare the clinical and cost-effectiveness of subcutaneous and oral methotrexate when used as first-line disease modifying anti-rheumatic drug in adults with rheumatoid arthritis and collect information about the acceptability of both routes of administration.

**Methods:**

MOOSE is an open-label, multi-centre, assessor-blinded, two-arm randomised controlled trial, with an internal feasibility assessment, economic evaluation and qualitative study. It is a secondary care-based trial, involving NHS hospital rheumatology clinics. Potentially eligible patients will be approached to participate around the time of their initial clinic visit. Eligible patients who consent will be randomised to either oral or subcutaneous methotrexate. Randomisation will be minimised by trial centre, 28-joint disease activity score, and disease duration. Interventions will be prescribed open-label with participants and clinicians aware of treatment allocated. Outcome assessors will be blinded to treatment allocation. Each participant will be in the trial for 52 weeks. The primary outcome is remission assessed at 24 weeks. Secondary outcomes include disease activity, quality of life, mental health and employment. A qualitative study will involve semi-structured interviews to analyse the acceptability of interventions. The health economic study will use healthcare utilisation data, quality of life data, and cost-estimates to model cost-effectiveness.

**Discussion:**

Whether to use subcutaneous or oral methotrexate first line for RA is an important question for patients and clinicians. MOOSE study will provide evidence on the clinical and cost-effectiveness of oral and subcutaneous routes of methotrexate administration to answer this important question.

**Trial registration:**

Prospectively registered with the International Standard Randomised Controlled Trial Number (ISRCTN) 14,403,521. Registered on 03 August 2023 https://doi.org/10.1186/ISRCTN14403521.

## **Administrative information**

Note: the numbers in curly brackets in this protocol refer to SPIRIT checklist item numbers. The order of the items has been modified to group similar items (see http://www.equator-network.org/reporting-guidelines/spirit-2013-statement-defining-standard-protocol-items-for-clinical-trials/).

**Table:** 

Title {1}	Methotrexate oral or subcutaneous for rheumatoid arthritis (MOOSE): study protocol for a multicentre randomised trial.
Trial registration {2a and 2b}	International Standard Randomised Controlled Trial Number (ISRCTN) 14403521
Protocol version {3}	V2.0 12-Jun-2024
Funding {4}	National Institute for Health and Care Research (NIHR) Health Technology Assessment Programme grant (NIHR 1006576)
Author details {5a}	The University of Nottingham Nottingham Clinical Trials Unit, The University of Nottingham University of Birmingham NIHR Southampton Clinical Research Facility, University Hospital of Southampton Clinical Research Facility Keele University Sheffield Centre of Health and Related Research, The University of Sheffield Department of Rheumatology, Bronglais Hospital, Hywel Dda University Health Board, Wales
Name and contact information for the trial sponsor {5b}	University of Nottingham sponsor@nottingham.ac.uk
Role of sponsor {5c}	The views expressed are those of the authors and not necessarily those of the NIHR or the Department of Health and Social Care. The funder and the sponsor had no input into the trial design, collection, analysis and interpretation of data, nor into the writing of the report or decision to submit the article for publication.

## Introduction

### Background and rationale {6a}

Rheumatoid arthritis (RA) is an inflammatory disease that most commonly affects the small joints of the hands and feet, causing considerable pain and functional impairment. It is a systemic disease and can cause a wide range of complications for patients, carers, the NHS and society. RA affects 0.7% of adults in the UK and can cause permanent joint damage and disability if not treated aggressively [1, 2]. Oral weekly methotrexate (≤ 25/week) has emerged as the first-line disease modifying anti rheumatic drug (DMARD) for the management of RA, with the proportion of patients treated with methotrexate increasing over time [3]. Self-administered subcutaneous injections are often used if there is gastrointestinal intolerance or suboptimal efficacy. According to our June 2020 survey of 33 UK rheumatologists, 14% offer methotrexate injections as a first-line treatment. Existing randomised controlled trials (RCTs) have suggested that first-line subcutaneous methotrexate has greater efficacy than first-line oral methotrexate [4–7], but these RCTs used a fixed dose of methotrexate, and did not employ a treat-to-target strategy with dose escalation of other drugs, as recommended in the National Institute for Health and Care Excellence (NICE) guidelines for the management of RA [4, 5]. Pharmacokinetic evidence supports the suggestion that subcutaneous methotrexate has greater efficacy, showing a plateau in bioavailability of oral methotrexate at doses > 15 mg/week, unlike for subcutaneous methotrexate [8]. A Canadian cohort study reported better control of disease activity with subcutaneous methotrexate compared to oral methotrexate but there was a large imbalance in starting dose, with 87% of participants starting subcutaneous methotrexate at 20–25 mg/week, whilst only 41% started oral methotrexate at these doses [9]. However, sub-cutaneous methotrexate can be painful to administer and cumbersome and are more expensive (£16.06 vs £0.75 for the 20 mg/week dose as per the British National Formulary (BNF) [10]. Cost-effectiveness analysis was not included in these studies. Evidence on effectiveness, tolerability and cost-effectiveness is required before injections can be recommended as a first-line treatment by NICE and British Society for Rheumatology (BSR).

### Objectives {7}

The MOOSE trial aims to compare the clinical and cost-effectiveness of subcutaneous and oral methotrexate in adults with RA and to collect information about the acceptability of both routes of methotrexate administration. The primary objective is to assess the effectiveness of a treat-to-target protocol using first-line subcutaneous methotrexate in comparison to oral methotrexate on remission of RA at 24 weeks. Secondary objectives include assessment of the effectiveness of a treat-to-target protocol using first-line subcutaneous methotrexate in comparison to oral methotrexate on disease activity, quality of life, mental health, patient acceptability of administration routes, progression to other DMARDs and employment.

### Trial design {8}

MOOSE is a pragmatic, prospective, assessor-blinded, randomised controlled superiority trial of subcutaneous methotrexate compared with oral methotrexate for patients diagnosed with RA. It is a parallel two-armed trial with participants randomised 1:1 to subcutaneous or oral methotrexate. It has an embedded Health Economic analysis and a parallel qualitative study on acceptability. A study within a trial (SWAT) will investigate whether inclusion of a trial information video (co-designed by the study’s Patient Advisory Group), available via QR code or URL within the Participant Information Sheet (PIS) increases recruitment over a PIS without the link.

## Methods: participants, interventions and outcomes

### Study setting {9}

386 participants will be recruited from at least 30 NHS rheumatology clinics across England, Scotland and Wales. To aid diversity, the selection of sites will include large cities and smaller, more rural, locations. A list of actively recruiting sites can be found on the trial website.

### Eligibility criteria {10}

The target population for the trial is methotrexate naïve adults with active RA.

#### Inclusion criteria

- Age ≥ 18 years.
- Meets American College of Rheumatology/European League Against Rheumatism (ACR/EULAR) classification criteria for RA [11].
- Active RA defined as at-least one swollen joint assessed by a rheumatologist.
- Willing to start treatment with either oral or subcutaneous methotrexate.
- 28-joint disease activity score with C-reactive protein (DAS-28-CRP) ≥ 2.6 (C-reactive protein (CRP) from prior clinic visit to be used to calculate this score at baseline visit).

#### Exclusion criteria

- RA previously treated with methotrexate or other disease modifying anti-rheumatic drugs. Patients treated with hydroxychloroquine for palindromic RA or autoantibody positive arthralgia are eligible.
- Psoriasis or other immune-mediated inflammatory conditions such as inflammatory bowel disease, ankylosing spondylitis, lupus, polymyalgia rheumatica or giant cell arteritis.
- Dementia, severe psychological disturbance i.e. mental health illness that makes receiving trial information and initial screening questions a stressful experience.
- Unable to give informed consent or comply with trial procedures.
- Cancer treatment i.e. surgery, radiotherapy, immunotherapy or chemotherapy, currently or in the last 12 months (current or past non-metastatic melanoma and skin cancer are eligible).
- Solid organ transplant on long-term daily prednisolone and/or other immunosuppressive treatments.
- Stage 4/5 chronic kidney disease, chronic liver disease (e.g. autoimmune hepatitis, primary sclerosing cholangitis, hepatitis B or C, cirrhosis).
- Contraindication to low-dose methotrexate.
- Pregnant or breast feeding.
- Planning to become pregnant or breast feed within the next 18 months.
- For men, intending to start a family within the next 18 months.
- Life expectancy less than 12 months

### Who will take informed consent? {26a}

Potential participants will be approached about the MOOSE trial by their clinical care team around the time of the patient’s first presentation to the rheumatology clinic. Upon return to clinic for the baseline visit, written informed consent will be taken by the investigator, or delegate after the patient has had the opportunity to ask any further questions. Patients considered eligible for methotrexate treatment will be approached, but no trial assessments will be completed prior to full trial consent at the baseline visit.

### Additional consent provisions for collection and use of participant data and biological specimens {26b}

Optional consent will be taken for (i) obtaining data from medical records for a 2-year follow-up, (ii) use of the participants mobile phone number to send questionnaire reminder text messages, and (iii) participants willing to be contacted about being interviewed for the qualitative study.

Consent to take part in the qualitative study will be taken separately for the 20 individuals selected to complete interviews regarding treatment acceptability. The participant will either complete the consent form for the qualitative study prior to the interview, or just prior to the interview the research team will take consent verbally and record the responses in an online form, prior to any treatment acceptability discussions. Verbal consent will be recorded for the purpose of monitoring the consent process.

Consent will be taken for blood samples additional to those required for clinical care, to be taken at baseline and week 24, and for the samples to be tested at the local hospital attended for treatment of rheumatoid arthritis. Sample collection, storage and destruction, and analysis will be completed as per usual practice in the participants local NHS hospital.

## Interventions

### Explanation for the choice of comparators {6b}

As the usual care first-line treatment for RA, oral methotrexate will be used in the comparator arm of the trial.

### Intervention description {11a}

The investigational medicinal product (IMP) for MOOSE, methotrexate, will be prescribed open-label, in either oral tablet or subcutaneous injectable form. The IMP is defined by its active substance only, and all authorised brands in the UK may be used.

Subcutaneous methotrexate will be used in the intervention group and will be prescribed as pre-filled injector pens or pre-filled syringes for self-administration.

Oral methotrexate in tablet form will be used in the comparator group.

All IMPs will be typically prescribed at an initial dose of 7.5 mg/week to 15 mg/week. Higher or lower starting doses may be chosen as clinically indicated or as per their usual practice. The dose may be increased gradually according to disease activity or tolerability but will not exceed a weekly dose of 25 mg/week. Both subcutaneous and oral methotrexate will be dispensed from the hospital or community pharmacy at the randomisation visit as per usual practice in that region, with folic acid, as per the BSR guidelines.

Standard NHS supplies will be used in accordance with their marketing authorisation. The allocated IMP will be dispensed to the trial participant in accordance with a prescription given by an authorised healthcare professional and labelled in accordance with the requirements of Schedule 5 to the Medicines for Human Use (SI 1994/31 94) (Marketing Authorisations, etc.) Regulations 1994 that apply in relation to relevant dispensed medicinal products.

### Criteria for discontinuing or modifying allocated interventions {11b}

If a participant is unwilling to increase the methotrexate dose, develops side-effects to methotrexate, or the maximum licensed dose of methotrexate is unable to achieve the treatment target of remission, alternate DMARDs may be prescribed as per the rheumatologist’s usual clinical practice. This may either be as sequential monotherapy or add on combination therapy depending on preferences of the participant and the rheumatologist. All DMARDs licenced for management of RA are permitted for use in this trial.

Participants may progress to biologic (b) and/or targeted synthetic (ts) DMARDs as per the latest NICE guidelines for the management of RA.

Participants may use physiotherapy, occupational therapy or any other therapy input at the discretion of their rheumatologist.

Participants randomised to subcutaneous (SC) methotrexate may switch to oral methotrexate or to another DMARD if there are any side-effects such as injection site reaction, inability to self-inject, or lack of efficacy, if the treating clinician feels that this is necessary, reflecting clinical practice.

Similarly, participants randomised to oral methotrexate will be able to switch to subcutaneous methotrexate or to any other DMARD, for side-effects such as gastro-intestinal intolerance or lack of efficacy if the treating clinician feels that this is necessary, reflecting clinical practice.

### Strategies to improve adherence to interventions {11c}

Adherence to methotrexate route of administration will not form part of the progression criteria for the trial since this is a pragmatic treat to target protocol that aims to reflect clinical practice but will be regularly monitored by the trial management group (TMG).

### Relevant concomitant care permitted or prohibited during the trial {11d}

Initial use of combination therapy (e.g. methotrexate plus hydroxychloroquine and/or sulfasalazine) will not be permitted. Patients already taking hydroxychloroquine for previous palindromic RA or antibody positive arthralgia will be allowed to continue hydroxychloroquine at the discretion of their rheumatologist.

Apart from the above restriction all concomitant medications will be used throughout the trial as per usual practice.

### Provisions for post-trial care {30}

When the trial ends, participants will continue to be treated by their usual care team, following local practices. Any changes in treatment will be decided by the participant and their rheumatologist.

### Outcomes {12}

The primary outcome is clinical remission of RA, defined as DAS-28-CRP < 2.6. This will be assessed at week 24. Secondary outcomes include remission defined as DAS-28-CRP < 2.6 at weeks 12 and 52, disease activity and response to treatment assessed by Clinical Disease Activity Index, Simplified Disease Activity Index, EULAR and ACR responses. Participant-reported outcomes include validated questionnaires to assess function, quality of life, mental health, and work productivity. See Table 1 for a full summary of secondary outcomes, and Table 2 for the SPIRIT figure of enrolment, interventions and assessments.

**Table 1:** Secondary outcomes

	**Outcome**	**Timepoints**
E1	Remission of RA (DAS-28-CRP)	12, 52 weeks
E2	Remission of RA (SDAI)	12, 24, 52 weeks
E3	Remission of RA (CDAI)	12, 24, 52 weeks
E4	Remission of RA (ACR/EULAR 2022 Boolean)	12, 24, 52 weeks
E5	Disease Activity of RA (CDAI)^a^	12, 24, 52 weeks
E6	Disease Activity of RA (SDAI)^a^	12, 24, 52 weeks
E7	Response to treatment (ACR20, ACR50, ACR70)	12, 24, 52 weeks
E8	Response to treatment (EULAR response criteria)	12, 24, 52 weeks
E9	DAS-28-CRP score	12, 24, 52 weeks
E10	SDAI score	12, 24, 52 weeks
E11	CDAI score	12, 24, 52 weeks
E12	Swollen joint count	12, 24, 52 weeks
E13	Tender joint count	12, 24, 52 weeks
E14	Patient global assessment (CDAI, SDAI question) (PGA)	4, 8, 12, 24, 52 weeks
E15	Physician global assessment (PhGA)	12, 24, 52 weeks
E16	Patient global health (ACR question) (PGH)	12, 24, 52 weeks
E17	CRP	12, 24, 52 weeks
E18	Patient pain	12, 24, 52 weeks
E19	Function (HAQ-DI)	12, 24, 52 weeks
E20	Fatigue (FACIT-F)	24, 52 weeks
E21	Anxiety (GAD-7)	24, 52 weeks
E22	Depression (PHQ-8)	24, 52 weeks
E23	Treatment acceptability (TFA)	4, 24, 52 weeks
E24	Beliefs about Medicines (BMQ)	4, 24, 52 weeks.
E25	EQ-5D-5L	12, 24, 52 weeks
E26	Quality of life (RA-QoL)	24, 52 weeks
E27	Work productivity and employment (WPAI)	24, 52 weeks
E28	Proportion of participants receiving corticosteroid(s)	12, 24, 52 weeks
E29	Proportion of participants who discontinue randomised treatment	12, 24, 52 weeks
E30	Time to discontinuation of randomised treatment	By 52 weeks
E31	Proportion of participants starting on any additional/alternative DMARDs	12, 24, 52 weeks
E32	Time to start on any additional/alternative DMARDs	By 52 weeks
E33	Proportion of participants starting a biologic drug	12, 24, 52 weeks
E34	Time to start of biologic drug	By 52 weeks
	**Qualitative outcomes**	
Q1	Treatment acceptability (interviews)	4-8 weeks, 24-32 weeks
	**Safety outcome**	
S1	Incidence of infection	4, 8, 12, 24, 52 weeks
S2	Incidence and severity of methotrexate side effects	4, 8, 12, 24, 52 weeks
S3	Incidence, type and severity of AEs	12, 24, 52 weeks
S4	Incidence of SAEs	52 weeks

^a^As remission, low, moderate, or high disease activity

**Table 2:**
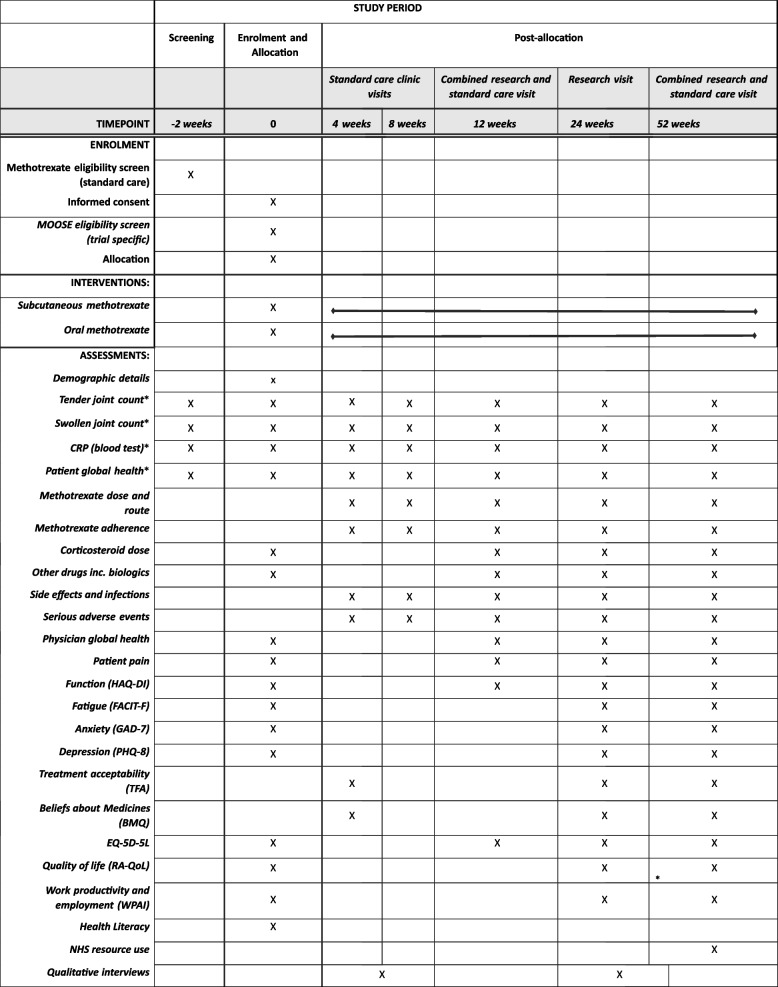
Enrolment, interventions and assessments

*These measurements will be used to compute remission, DAS-28-CRP score, Clinical Disease Activity Index, Simplified Disease Activity Index, EULAR, and ACR responses

#### Safety outcomes

The incidence of the following infections will be collected at 4, 8, 12, 24 and 52 weeks using patient questionnaires.

- Herpes zoster (shingles).
- Urinary tract infection requiring antibiotics.
- Chest infection or pneumonia requiring antibiotics.
- Skin or soft tissue infection (including cellulitis) requiring antibiotics.
- COVID-19 (must have had a positive PCR or lateral flow test).

The following methotrexate side effects will be collected at 4, 8, 12, 24 and 52 weeks using patient questionnaires:

- Nausea.
- Abdominal pain.
- Bloating of the abdomen.
- Diarrhoea.
- Vomiting.
- Mucositis (oral).
- Injection site reaction (methotrexate injection only).

The severity of these side effects based on Common Toxicity Criteria (CTC) criteria will also be collected. Additional adverse events (AEs) will be collected at each clinic visit. Discontinuation of methotrexate due to safety or tolerability concerns will also be recorded as part of the eCRF and reviewed monthly by the TMG. The Data Monitoring Committee (DMC) will review safety and tolerability data annually, or more regularly at the request of either the TMG or DMC.

Blood tests to screen for idiosyncratic blood, liver, or kidney damage will be taken as part of routine safety monitoring for the duration of treatment. Abnormal results relating to leucocyte count, neutrophil count, platelet count, alanine transaminase (ALT) level, aspartate transaminase (AST) level, and creatine level will be recorded in the eCRF. Additional results of concern will be reported as adverse events.

Treatment acceptability outcomes identify perceptions of changes in acceptability of interventions and influences of change within case (each person) and in each intervention group (cross-case). Additionally, using the Beliefs about Medicines Questionnaire, a Necessity-Concerns Differential will be calculated. The qualitative analysis will be merged with the quantitative analysis (from the theoretical framework of acceptability questionnaire (TFA)) to provide enhanced understanding of treatment acceptability and adherence and to help explain any variations in trial outcomes [12].

A mathematical model will be developed to estimate the cost-effectiveness of subcutaneous methotrexate compared with oral methotrexate. The model will take account of the results observed in the pragmatic trial, particularly those associated with costs to the NHS, EQ-5D-5L values, discontinuation rates and remission rates. The analyses will be in line with the NICE reference case, estimating a cost per quality-adjusted life year (QALY) gained for the more efficacious treatment from a probabilistic analysis [13].

The SWAT primary outcome will be the proportion of patients given a PIS who are consented at each site. Secondary outcomes include the proportion of participants providing primary outcome data (at 24 weeks), and the proportion of participants remaining in the trials at 52 weeks.

#### Summary of secondary outcomes

The summary of secondary outcomes is shown in Table 1.

#### Enrolment, interventions and assessments

The SPIRIT figure is shown in Table 2.

### Participant timeline {13}

**Fig. 1:**
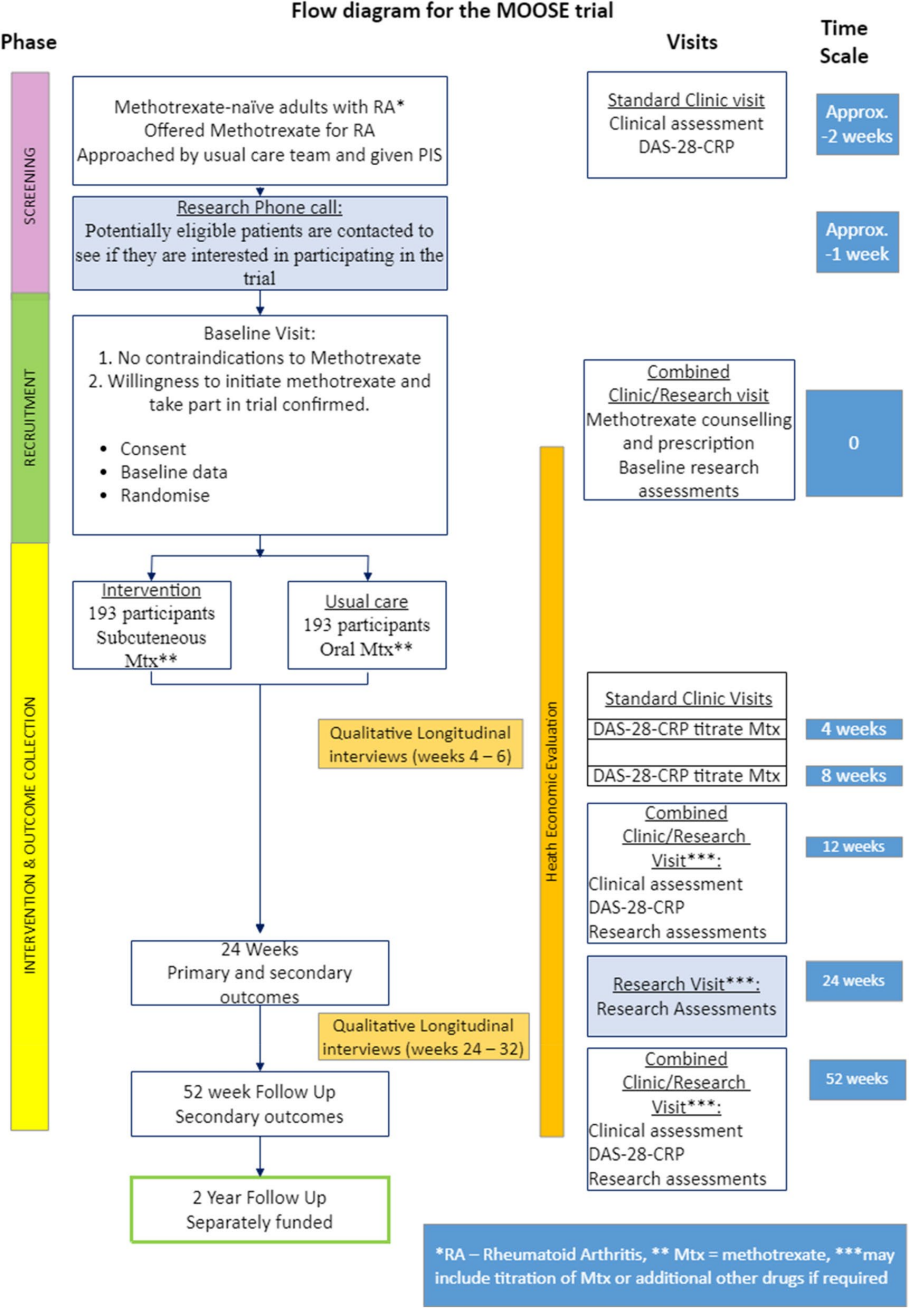
Patient pathway

The participant timeline is detailed in Fig. 1.

Patients will be approached at a screening visit around the time of the first presentation at clinic where they are offered methotrexate as a treatment. This is a routine visit where they will have clinical assessments, any initial treatments needed e.g. corticosteroids, blood tests including full blood count, liver function test, urea electrolytes and creatinine, inflammation markers CRP and erythrocyte sedimentation rate (ESR), and imaging as per usual practice. In accordance with standard practice, auto-antibody (rheumatoid factor (RF), and anti-CCP antibody), should be checked at the screening visit if not already previously checked. Safety blood tests (e.g. screening for prior viral infections) will also be checked at the screening visit. Approach can be followed up by a telephone call prior to the baseline visit to gauge interest.

The baseline visit will take place approximately 2 weeks after screening, depending on local practice and capacity. If there are no contraindications to methotrexate and the patient is willing to initiate methotrexate treatment, consent will be taken, and baseline assessments will be done. DAS-28-CRP scores will be calculated using joint counts and the CRP blood test taken at the screening visit, and demographic details will be provided. The patient will be asked to complete the baseline questionnaire booklet. Methotrexate counselling should be provided for both subcutaneous and oral methotrexate, or if not possible at this stage it should be done immediately after the treatment allocation is known. Eligibility will be confirmed by a medically qualified doctor, and the rheumatologist will determine the initial dose of methotrexate depending on their prescribing practice. The patient will be randomised to receive either oral or subcutaneous methotrexate, and prescribed the dosage decided prior to randomisation.

Dose escalation visits will take place at 2–4 week intervals after taking the first dose of methotrexate.

Follow-up visits will take place at weeks 12, 24 and 52. Tender joint count (TJC), swollen joint count (SJC) and physician global assessment (PhGA) will be made by an assessor blinded to the treatment allocation. During these follow-up visits the participant will have a CRP blood test, complete the questionnaire booklet, and dose may be escalated or other concomitant medications or DMARDs prescribed.

A 2-year follow-up may take place using the participants’ medical records. There will be no additional clinic visits at 2 years.

At weeks 4–6, and again at weeks 24–32, a small number of participants, 10 from each intervention group, will be invited to participate in the qualitative study interviews.

### Sample size {14}

The sample size is based on the primary outcome—the proportion of participants showing remission defined as a DAS-28-CRP < 2.6, 24 weeks from randomisation. A survey of 33 rheumatologists in June 2020 indicated that 13 (39%) and 29 (89%) would prescribe subcutaneous methotrexate if it increased remission by 15% or 20%, respectively. Given this marked difference, the trial was powered to detect an absolute difference of 17.5% between oral and subcutaneous methotrexate. Assuming that 30% of participants in the oral methotrexate arm were in remission at week 24 [14], 173 participants would be required in each arm to detect a difference of 17.5% (i.e. 47.5% of participants in the subcutaneous arm in remission), with 90% power, and a 2-sided alpha of = 5%. Assuming 10% loss to follow-up at 24 weeks, 386 participants should be randomised. Since the primary objective of the trial is to assess the relative effectiveness of first-line subcutaneous versus oral methotrexate, the primary comparison will be as randomised (i.e. intention to treat). Therefore, no additional adjustments to the sample size are necessary. Power calculations were performed using PASS v12 (NCSS).

### Recruitment {15}

Strategies to promote the trial include a poster for use in clinic areas and a postcard to hand out to any interested parties. A trial website will contain information for both patients and recruiting staff, including regular updates on recruitment figures.

At the initial presentation visit to the rheumatology clinic, potential participants will be given the PIS. Site staff will have the opportunity to post or email a PIS to patients who were not approached in the clinic. They will also be able to make a telephone call to the patient to discuss the trial and gauge interest.

Participants will be offered two £25 vouchers as a thank you for their involvement and to help cover any additional transport costs they may experience. These will be given after baseline and 24-week visits.

Sites randomised to SWAT intervention group 1 will use the video PIS, which has a QR code link to an information video, showing the trial in animated format, and a nurse demonstration of using the subcutaneous methotrexate injection device. At the interim analysis stage, if there is sufficient evidence of a difference in consent rates between the two SWAT arms in favour of the SWAT intervention, the video SWAT will be rolled out across all sites with the aim of improving recruitment across sites.

Site recruitment targets will be set according to size and capacity of the local team. Recruitment will not be capped; and all involved NHS trusts will be given the opportunity to over recruit up to the point of the overall target recruitment figure of 386 being met. The MOOSE patient advisory group, consisting of RA patients and/or carers will be involved at key stages throughout the trial, including contribution to the development of recruitment strategies, data collection and retention. Scheduled meetings will take place, but there will also be capacity to meet on an ad-hoc basis as-and-when issues with recruitment arise.

## Assignment of interventions: allocation

### Sequence generation {16a}

Eligible patients who consent will be allocated to receive either subcutaneous methotrexate or oral methotrexate on a 1:1 ratio. Treatment will be assigned randomly using a minimisation algorithm balancing by trial recruiting centre, DAS-28-CRP (< 5.1 and ≥ 5.1) and disease duration (< 4 months, 4–12 months, and > 12 months). These variables are selected due to their likely association with the primary outcome.

### Concealment mechanism {16b}

Allocation will be concealed using a web-based randomisation system developed and maintained by the Nottingham Clinical Trials Unit (NCTU) and hosted on a secure server, accessed via a secure website.

### Implementation {16c}

The site Principal Investigator (PI), PI delegate or research nurse conducting the patient visit will enrol the participant on the MOOSE Research Electronic Data Capture (REDCap) database system. Randomisation is integrated within the REDCap database. When all baseline data has been completed, and randomisation requested, the database provides details of treatment allocation on the randomisation form.

## Assignment of interventions: blinding

### Who will be blinded {17a}

Interventions are open-label therefore participants and their care providers including rheumatologist will not be blinded to the treatment allocation. Disease activity (DAS-28-CRP) assessors will be blinded to the treatment allocation throughout the trial. This may be a research or usual care team member. The trial statistician will be blinded and will not have access to individual participant data until after the database has been locked. Adherence to allocated treatment will be provided by an unblinded independent statistician, who will also provide any unblinded disaggregated data for the DMC. The TSC will be blinded to treatment allocation unless specifically recommended by the DMC. The qualitative researchers will not be blinded to the participants route of methotrexate allocation.

The Chief Investigator (CI) will be partially blinded, only having access to the unblinded data for participants randomised at their site, or where serious adverse event (SAE) review is determined to be related to the trial treatment. The TMG will be partially blinded to treatment allocation, with only the trial management and data management staff having access to participant data that may be unblinding.

### Procedure for unblinding if needed {17b}

Only researchers assessing disease activity (DAS-28-CRP) will be blinded to treatment allocation. Interventions will be open-label and both participants and research clinicians will be aware of the treatment allocation; therefore, there is no requirement for blind-breaking procedures.

## Data collection and management

### Plans for assessment and collection of outcomes {18a}

Trial outcome data from clinic visits will be collected in the REDCap database. Site staff will enter baseline data and assessments, and follow-up assessment data directly into REDCap. Blinded assessment data is collected on a paper CRF and later transcribed to the eCRF by an unblinded member of the research team. Participant questionnaires at weeks 4 and 8 are either entered directly to the database by participants who receive an individual link by email, or by post if preferred and transcribed to the eCRF by NCTU staff.

Qualitative data collection will involve semi-structured interviews in both arms. Maximum variance sampling will ensure patient diversity e.g. age, gender, ethnicity, health literacy, perceptions of acceptability. We aim to interview approximately 10 participants in each arm, depending on data saturation. All those interviewed at 4–8 weeks will be invited to another interview between 24 and 32 weeks.

### Plans to promote participant retention and complete follow-up {18b}

Participant research visits have been aligned where possible to coincide with standard care clinic visits so as not to burden the participant with additional clinic visits. Discussions with the study’s public co-applicant (MB) and our Patient Advisory Group has informed our plans to promote participant retention and follow-up. DAS-28-CRP assessments are in line with usual care, and participants are requested to complete the patient-reported outcome questionnaires whilst in clinic. Follow-up is continued regardless of whether the participant is adherent to trial intervention and is encouraged by reiterating the importance of the follow-up visit both to site staff and participants, by sending monthly newsletters to site staff and quarterly participant newsletters. The week 24 visit is an additional research visit, and at this time point participants receive a £25 voucher. Participants who wish to discontinue are given the option to partially remain in the study. This may be in the form of attending some, but not all, follow-up visits or by completing fewer questionnaires. All data collected before discontinuation will remain in the study.

Text messages and email reminders will be sent to participants to prompt them to complete the 4 and 8 week questionnaires, followed by a telephone call if required.

### Data management {19}

The Data Management Plan (DMP) will include the agreed validation specification, roles and responsibilities for the trial data and user access. The trial database, REDCap, is a validated secure web-based platform which allows for data tracking via date stamped audit logs. MOOSE participants will be identified on REDCap only by a unique participant identifier (their trial/participant ID) to protect from bias and ensure confidentiality.

Measures to improve data quality include warnings flagging database entries that are outside of the protocol parameters, for outcome measures including DAS-28-CRP components. Data reported on each eCRF will be checked for missing data or discrepancies. Decisions on how to treat anomalous data will be made by members of the TMG blinded to allocations and documented in the DMP and/or Statistical Analysis Plan (SAP); where required.

#### Qualitative data management

The contact details of those willing to participate in the interview study will be shared with the qualitative researcher at Keele University. The interview will be digitally audio/video-recorded, according to participant preference, and the digital file saved with the interview recordings and labelled with the participants’ unique ID number. The recordings will be shared securely with an approved transcription company for the purposes of transcription. Data will be held on Keele University’s secure data servers. Access to the MOOSE qualitative data will be restricted to named authorised individuals.

### Confidentiality {27}

All trial staff and investigators will endeavour to protect the rights of the trial’s participants to privacy and informed consent, and will adhere to the Data Protection Act, 2018. The CRF will only collect the minimum required information for the purposes of the trial. Access to the CRF will be limited to the trial staff and investigators and relevant regulatory authorities. Participant data will be held securely and password protected, with access restricted by user identifiers. Identifiable information will be stored within a restricted channel of the REDCap database, and limited access allowed for the purpose of questionnaire and treatment acceptability interview communication. Information about the trial in the participant’s medical records will be treated confidentially in the same way as all other confidential medical information.

Individual participant medical information obtained as a result of this trial are considered confidential and disclosure to third parties is prohibited except for the need to disclose such medical information to the participant’s medical team and appropriate medical personnel responsible for the participant’s welfare. If information is disclosed during the trial that could pose a risk of harm to the participant or others, the researcher will discuss this with the CI and where appropriate report accordingly.

Data generated as a result of this trial will be available for inspection on request by the participating physicians, the University of Nottingham representatives, the Research Ethics Committee (REC), local NHS R&D departments and the regulatory authorities.

### Plans for collection, laboratory evaluation and storage of biological specimens for genetic or molecular analysis in this trial/future use {33}

All blood tests, including those taken at standard care clinic visits, and those taken at research visits, will be labelled, analysed and destroyed as per the local NHS hospital policy.

## Statistical methods

### Statistical methods for primary and secondary outcomes {20a}

The analysis and presentation of the trial results will be in accordance with the CONSORT guidelines and a full SAP will be developed prior to database lock. The primary objective of the trial is to determine the effectiveness of first-line subcutaneous versus oral methotrexate and as such, the principal approach to our primary comparative analysis will be to analyse as randomised without imputation of missing data, with due emphasis being placed on the confidence intervals for the between arm comparisons. Sensitivity and secondary analyses will be considered supportive to the primary. Characteristics and baseline data of randomised participants in the two trial arms at baseline will be described, using appropriate descriptive statistics.

The evaluation of the primary outcome will be performed using a mixed effects model for binary outcomes that includes study centre and disease duration as per the minimisation, and baseline DAS-28-CRP calculated using the CRP value obtained on the day of randomisation.

The primary estimands comparing the proportion of participants in remission at 24 weeks between those randomised to first-line subcutaneous methotrexate and first-line oral methotrexate, regardless of whether participants do not take or discontinue assigned treatment, or start a new treatment as add on or replacement therapy, will be the adjusted risk difference and 95% confidence interval.

#### Definition of populations analysed

Intention to treat dataset: All randomised participants are summarised/analysed according to their randomised treatment irrespective of the treatment(s) they received. This is the primary dataset to be used in both the effectiveness and the safety analyses.

Safety dataset: All randomised participants are summarised according to the treatment they receive, irrespective of their randomised allocation. This dataset may be used for sensitivity analyses.

Secondary outcomes will be analysed using appropriate regression models that include site and disease duration as recorded for minimisation, and DAS-28-CRP calculated using all components as collected at baseline, and baseline values of that outcome if measured and will be as randomised without imputation of missing data, unless otherwise indicated in the SAP.

### Interim analyses {21b}

No formal interim analyses are planned for the main trial. A feasibility assessment has been built into the trial in the form of an internal pilot phase examining recruitment and retention. The stop–go criteria (shown in Table 3 below) will be used to determine the progression of the trial recruitment 9 and 15 months after the first participant is randomised. Recruitment will be assessed against the overall recruitment target at 9 and 15 months. Retention will be reviewed at 15 months and a decision made based on the proportion of participants who have withdrawn from the trial (trial follow-up not allocated treatment) at or before the 24 week follow-up visit. The above criteria to aid decision making about progression of the trial has been proposed by the trial team and agreed with the TMG, and funder (NIHR). The final agreement on whether the trial should stop or continue will take place after discussion with NIHR. Adherence to methotrexate route of administration will not form part of the progression criteria for the trial since this is a pragmatic treat to target protocol but will be regularly monitored by the TMG.

**Table 3:**
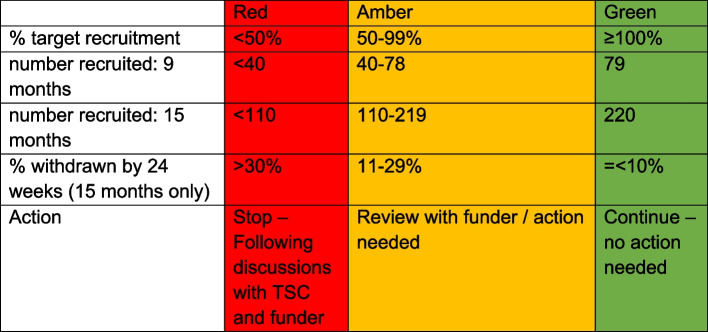
Recruitment and retention progression guidance for internal pilot

#### MOOSE stop/go criteria

The MOOSE stop/go criteria is shown in Table 3.

The SWAT interim analysis to compare the proportions of participants consenting in the two intervention groups (those with PISs with the QR code to the information video and those with PISs which do not have the QR code) will be performed at 9 and then potentially at 15 months after the first participant is randomised to determine whether there is a greater proportion consenting in either of the intervention groups.

Where there is a notable difference in consent rates (as defined in the SWAT SAP) at 9 months, the PIS which is associated with the higher consent rate will be adopted for the remainder of the trial. If there is no difference, an additional interim analysis will be performed at 15 months. If a notable difference is observed at this point, the PIS which is associated with the higher consent rate will be adopted for the remainder of the trial. Where no notable difference is observed, both PISs will be used until the end of the trial.

### Methods for additional analyses (e.g. subgroup analyses) {20b}

The comparison of first-line subcutaneous and oral methotrexate on the primary outcome only will be performed in subgroups according to disease duration (less-than 4 months, 4–12 months, and more than 12 months), auto-antibody status (only RF positive, only anti-CCP positive, dual sero-positive, seronegative), body mass index (≥ 30 vs. < 30 kg/m2) and smoking status (current-smoker vs. not-currently smoking at the screening visit). The interpretation of any subgroup effect will be based on interaction tests (i.e. evidence of differential treatment effects in the different subgroups). It is acknowledged that these investigations will not be adequately powered.

Treatment acceptability interviews will be transcribed verbatim and analysed thematically using a framework approach [15, 16]. Following data familiarisation, a thematic framework will be developed using inductive and deductive coding [16]. For longitudinal analysis, summaries of each participant’s data [17, 18] will be used to identify perceptions of changes in acceptability of interventions and influences of change within case (each person) and in each intervention group (cross-case). Using the Beliefs about Medicines Questionnaire, a Necessity-Concerns Differential will be calculated. This score will provide a numerical indicator of how participants judge their personal need for different formulations of methotrexate relative to their concerns about the potential negative effects of taking methotrexate. Interpretation of data will be discussed with the patient advisory group and with researchers from different professional backgrounds (e.g. rheumatology, health services research), improving the trustworthiness of analysis [16, 19].

For the health economic analysis, both the during trial and future extrapolation models, the incremental cost-effectiveness ratio (ICER) estimated from probabilistic analyses will be calculated following guidance published by NICE [13]. The uncertainty in these ICERs will be explored, using seemingly unrelated regression for the first model [20] and for the second by estimating the confidence interval in the calculated ICERs presented, alongside an estimate in the true underlying uncertainty using a percentile approach [21]. To visualise the uncertainty, cost-effectiveness planes and cost-effectiveness acceptability curves will be provided. Comprehensive scenario/sensitivity analyses will be undertaken to explore the robustness of the results to changes in the values of key parameters (such as the projected use of biologic disease modifying anti-rheumatic drugs (e.g. anti-TNF agents), requirement for surgery and utility values for patients) to alternative plausible values, and by the inclusion of monetised values for absenteeism and presenteeism. If appropriate, value of information analyses will be conducted to show whether there is an incentive to collect further information, and on which parameters [22, 23]. The results from the value of information analyses will indicate the maximum cost of research to reduce decision uncertainty and will indicate whether further research would be seen as cost-effective.

### Methods in analysis to handle protocol non-adherence and any statistical methods to handle missing data {20c}

It is known that some participants may have their first-line therapy discontinued due to lack of efficacy or tolerability or have additional therapies added to that first-line therapy. 55 of the 151 participants randomised to the intensive treatment arm in the CAMERA study swapped from oral to subcutaneous methotrexate injections for either intolerance or lack of efficacy and 20% participants prescribed oral methotrexate in the CATCH cohort swapped from oral to subcutaneous and 3% from sub-cutaneous to oral [9, 24]. In addition, participants may not take their medications as directed and may receive rescue medications on a planned temporary basis (e.g. corticosteroids to treat RA flares). We will therefore collect data to allow us to characterise and investigate such intercurrent events and estimate the efficacy of the two treatments despite the pragmatic trial design.

The primary analyses will not use any imputation techniques. However, the SAP will document where methods to address missing data (for example multiple imputation in a sensitivity analysis) will be used.

### Plans to give access to the full protocol, participant-level data and statistical code {31c}

De-identified participant data and associated meta-data will be made available, upon request, in accordance with the NCTU standard operating procedures following the publication of the trial results.

## Oversight and monitoring

### Composition of the coordinating centre and trial steering committee {5d}

The MOOSE Trial team at the NCTU will have oversight of day-to-day activities of the trial. The trial team will check incoming data for adherence to with the protocol and treatment arm, data consistency and widespread missing data, as per the trial monitoring plan. The TMG comprising the full co-applicant team, including a member of the patient advisory group, and NCTU staff will meet on a monthly basis and will be responsible for the general management of the trial. Independant trial oversight will be provided by the TSC who will meet 6 monthly or in line with feasibility assessments to monitor progress against targets, and advise.

### Composition of the data monitoring committee, its role and reporting structure {21a}

An independent DMC will meet 6 monthly or in line with feasibility assessments. They will also be responsible for monitoring safety, and data for consistency with the sample size assumptions. Emergency meetings may be convened if a safety issue is identified. The DMC will report directly to the TSC, who will convey the findings of the DMC to funder, sponsor, and regulatory authorities as applicable.

### Adverse event reporting and harms {22}

As the safety profiles of the IMPs used in this trial are well characterised, we will adopt a targeted approach to adverse event (AE) reporting. AEs due to disease progression will be excluded from expedited reporting. Known treatment-related AEs will be collected as part of the participant questionnaires. Additional AEs reported by participants will be collected and reported on the adverse event log. AEs will be coded using the Medical Dictionary for Regulatory Activities (MedDRA) as per NCTU standard practice.

Common toxicity criteria for adverse events: Known AEs to be collected to assess tolerability of randomised treatment are, abdominal pain, nausea, vomiting, diarrhoea, bloating, oral mucositis and injection site reaction.

Infection information will be collected as part of each follow-up questionnaire relating to recent diagnoses of herpes zoster (shingles), urinary tract infections requiring antibiotics, chest infections/pneumonia requiring antibiotics, cellulitis requiring antibiotics and COVID-19.

Blood test results will be reviewed as part of usual care to identify abnormal results. Abnormal results relating to leucocyte count, neutrophil count, platelet count, ALT level, AST level, and creatine level will be recorded in the CRF. Abnormal results meeting the seriousness criteria will be reported to the NCTU as an SAE.

As methotrexate is a long-established drug, no safety signal are expected in these blood tests. Both oral and subcutaneous routes are well established modes of administering methotrexate in the treatment of RA. Abnormalities will be managed by the local usual care team and any SAE and/or suspected, unexpected serious adverse reaction (SUSAR) data will be reported to the DMC at their annual meeting.

Where a site becomes aware of a pregnant participant during the trial a Notification of Pregnancy form will be completed and returned to NCTU, and the participants GP notified of the pregnancy. Pregnant participants must stop methotrexate and be followed up as part of their routine care.

### Frequency and plans for auditing trial conduct {23}

The NCTU Quality Assurance (QA) team will carry out systems and trial audits as part of the NCTU risk-adapted annual audit programme. Should this trial be selected for audit, an audit report shall be issued to the Trial Manager and can be disseminated to the appropriate committees should this be appropriate. Where monitoring has identified the need for a site audit, or this is requested of the TMG/TSC, this shall be carried out by a trained member of NCTU staff.

### Plans for communicating important protocol amendments to relevant parties (e.g. trial participants, ethical committees) {25}

All amendments made to the trial protocol will undergo review and approval by the Sponsor, REC, Medicines and Healthcare products Regulatory Agency (MHRA) and Health Research Authority (HRA) as required, prior to implementation. Updated versions of the protocol will be shared with recruiting centres via email and uploaded to the trial website. Any substantial changes to patient information will be communicated to participants by their recruiting site.

### Dissemination plans {31a}

Trial results will be reported in a peer reviewed journal, published on relevant websites and presented at conferences. Participants will be notified of the results in an end of trial letter and will be able to view the results on the website. Participants will not be identified in any publications or presentations. Publications and presentations (other than the protocol) will typically happen after the end of the trial.

## Discussion

The MOOSE study is designed to investigate whether low-dose weekly methotrexate (≤ 25 mg/week) administered as a subcutaneous injection is more effective and cost-effective compared to low-dose weekly methotrexate (≤ 25 mg/week) administered as an oral tablet when used as first DMARD in patients diagnosed with RA. It will also evaluate the acceptability of both routes of administration of methotrexate. It has broad eligibility criteria, uses a treat-to-target approach, and there are very few restrictions on the use of concomitant disease modifying/glucocorticoid sparing therapies meaning the study results will be applicable to the management of RA in the real-world setting. Nevertheless, this is an open label study and any bias from this will be minimised by using a blinded outcome assessor. The relatively large sample size will allow us to conduct several a priori subgroup analyses. We anticipate that the results of this study will inform treatment decisions around the route of administration of low-dose weekly methotrexate in the treatment of RA.

Results of the MOOSE study will inform national and international treatment recommendations.

### Trial status

MOOSE is in the recruitment phase. The current protocol is version 2.0 12-Jun-2024. Recruitment commenced Sept 2023 and is expected to end May 2025.

## Data Availability

Data sharing is not applicable to this article as no datasets were generated or analysed in preparation of this publication. De-identified data will be made available upon request, in accordance with the NCTU standard operating procedures following publication of the trial results.

## Abbreviations

RA: Rheumatoid arthritis
NHS: National Health Service
CRP: C-reactive protein
DAS-28-CRP: 28-joint disease activity score with C-reactive protein
ACR: American College of Rheumatology
EULAR: European League Against Rheumatism
CTC: Common Toxicity Criteria
ESR: Erythrocyte sedimentation rate
DMARD: Disease modifying anti rheumatic drug
QALY: Quality-adjusted life year
SWAT: Study within a trial
PIS: Participant Information Sheet
IMP: Investigational medicinal product
TMG: Trial management group
TSC: Trial Steering Committee
DMC: Data Monitoring Committee
PI: Principal Investigator
DMP: Data Management Plan
TJC: Tender joint count
SJC: Swollen joint count
PhGA: Physician global assessment
CI: Chief Investigator
REDCap: Research Electronic Data Capture
SAP: Statistical Analysis Plan
ICER: Incremental cost-effectiveness ratio
AE: Adverse event
SAE: Serious adverse event
GCP: Good Clinical Practice
REC: Research Ethics Committee
SUSAR: Suspected, unexpected serious adverse reaction
QA: Quality Assurance
MHRA: Medicines and Healthcare products Regulatory Agency
HRA: Health Research Authority
NICE: National Institute for Health and Care Excellence
BNF: British National Formulary
